# Disc hemorrhage following peripapillary retinoschisis in glaucoma: a case report

**DOI:** 10.1186/s12886-021-02010-5

**Published:** 2021-06-07

**Authors:** Won June Lee, Mincheol Seong

**Affiliations:** 1 Department of Ophthalmology, Hanyang University College of Medicine, Seoul, Korea; 2 Department of Ophthalmology, Hanyang University Seoul Hospital, Seoul, Korea; 3 Department of Ophthalmology, Hanyang University Guri Hospital, Kyougchun-ro 153, Guri-si, Gyeonggi-do Guri, Korea

**Keywords:** Glaucoma, Peripapillary retinoschisis, Disc hemorrhage

## Abstract

**Background:**

Disc hemorrhage (DH) is an important factor often associated with the development and especially progression of glaucoma. In contrast, some studies have reported peripapillary retinoschisis in glaucoma, but it is not recognized as a pathognomonic finding, and opinions on the clinical significance of retinoschisis are not consistent. Here,we present the case of DH following peripapillary retinoschisis in the same area within the same glaucomatous eye.

**Case presentation:**

A 70-year-old man with high intraocular pressure (IOP) was referred to the glaucoma clinic. At the time of the baseline study, the IOP was 30mmHg, and peripapillary retinoschisis was discovered at 7 o’clock on the periphery of the optic nerve with swept-source optical coherence tomography. Accompanying retinal nerve fiber layer defect were manifest in the inferotemporal part with red-free fundus photography. Under the impression of open-angle glaucoma, we prescribe latanoprost ophthalmic solution. Eight months later, the IOP was 17mmHg, and the peripapillary retinoschisis had disappeared. DH was observed in the inferotemporal area in the same direction as that of the previous peripapillary retinoschisis.

**Conclusions:**

The case presented here are the first to report on the relationship between peripapillary retinoschisis and DH. Hopefully future studies will reveal the actual connection between peripapillary retinoschisis and DH.

## Background

Glaucoma is a progressive optic neuropathy that accompanies structural changes in the optic nerve head and consequent visual field defects. Structural changes in the optic nerve head, such as damage to the neuro-retinal rim, deepening of disc cupping, and peripapillary retinal nerve fiber layer (RNFL) bundle defects, are well known [1]. Disc hemorrhage (DH) is widely known as a worse prognostic factor in glaucoma, and its relationship with the lamina cribrosa (LC) defect has also been reported [2]. In contrast, some studies have reported peripapillary retinoschisis in glaucoma, but it is not recognized as a pathognomonic finding, and opinions on the exact cause or clinical significance of retinoschisis are not consistent. Glaucoma-associated peripapillary retinoschisis often stabilises or spontaneously resolves [3–5]; however, some studies have reported that peripapillary retinoschisis is associated with rapid glaucoma progression [6, 7]. There have been no reports of these two findings in the same eye and a relationship between DH and peripapillary retinoschisis. We report a case of peripapillary retinoschisis preceding DH.

## Case presentation

**Fig. 1:**
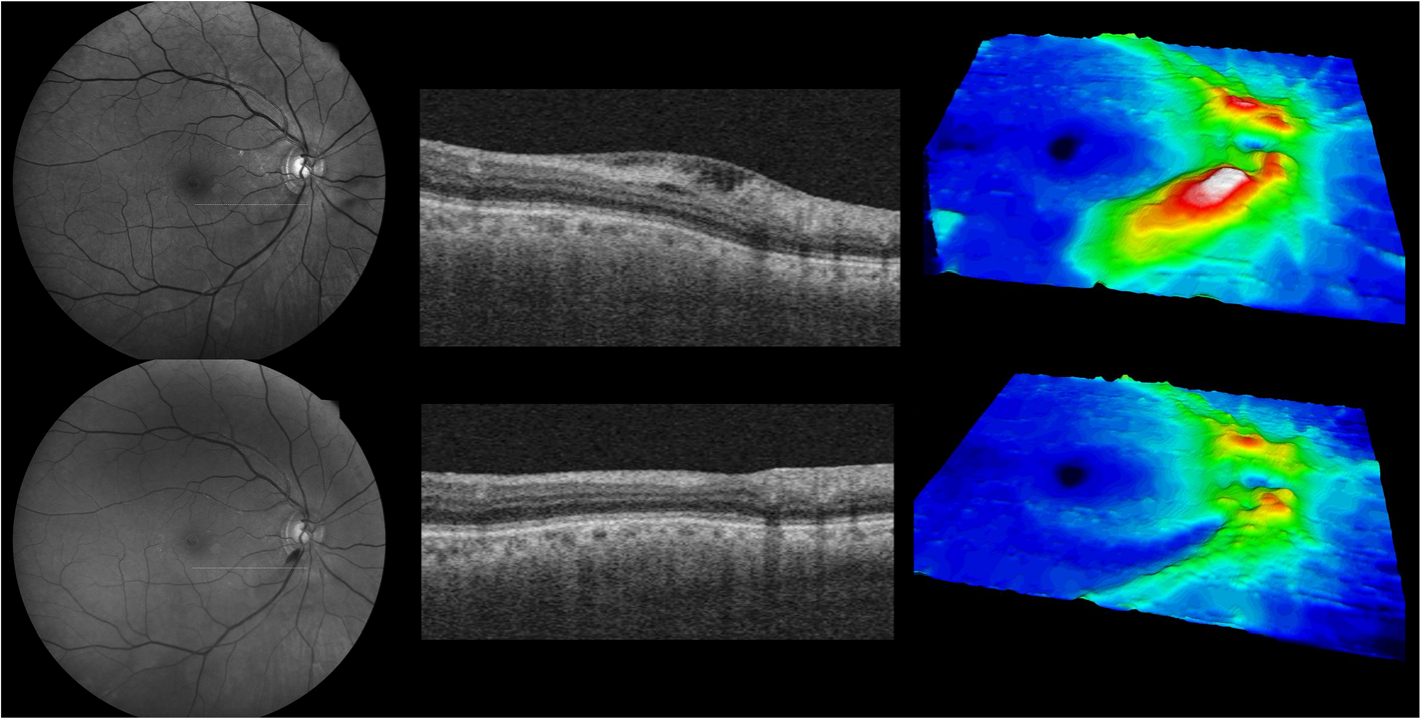
Serial red-free fundus photographs (first column), swept-source optical coherence tomography images (second column), and wide-field retinal nerve fiber layer thickness map images (third column) of the right eye of a 70-year-old man with open-angle glaucoma. Peripapillary retinoschisis was observed at the inferotemporal area of the optic disc (upper row). Eight months after the baseline image was taken, a disc hemorrhage was observed in the inferotemporal area of the right eye in the same direction as that of the peripapillary retinoschisis (lower row)

A 70-year-old man with high intraocular pressure (IOP) was referred to the glaucoma clinic. At the time of the baseline study, the IOP was 30 mmHg. and peripapillary retinoschisis was discovered at 7 o’clock at the periphery of the optic nerve with swept-source optical coherence tomography (OCT). Accompanying RNFL defects were observed in the inferotemporal part with red-free fundus photography (Fig. 1, upper row). There was no peripheral anterior synechiae and widely opened angle was observed on gonioscopic examination. Based on the suspicion of open-angle glaucoma, we prescribed a latanoprost ophthalmic solution. The IOP normalized, and the patient was regularly monitored while receiving medications. Eight months later, the IOP was 17 mmHg, and the peripapillary retinoschisis had disappeared. DH was observed in the inferotemporal area in the same direction as the previous peripapillary retinoschisis (Fig. 1, lower row).

**Fig. 2:**
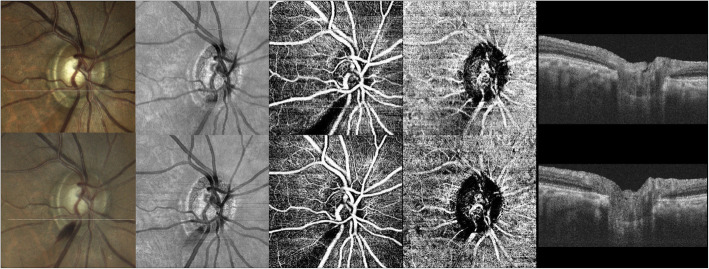
Serial disc photographs (first column), en-face images (second column), superficial capillary plexus of OCT-angiography (third column), deep choroidal layer of OCT-angiography (fourth column), and swept-source optical coherence tomography images (fifth column) of the optic disc of a 70-year-old man with open-angle glaucoma. Morphological changes in LC, including LC pore or LC defects, were not detected at baseline (upper row) and 8 months after the baseline (lower row). The decrease in vessel density of the superficial layer along with the RNFL defect is stationary, and the inferotemporal choroidal vascular dropout has not progressed

To evaluate the structural changes and blood flow status of the optic nerve head, multimodal imaging, including en-face image and OCT-angiography, was performed (Fig. 2). Morphological changes in the LC, including LC pores and defects, were not detected at baseline or during follow-up. A decrease in the vessel density of the superficial layer along with the RNFL defect was stationary, and the inferotemporal choroidal vascular dropout did not progress. There were no signs of deep vascular structure associated with the subretinal neovascular membrane both on peripapillary and macular OCT-angiography.

## Discussion and conclusions

With advances in OCT technology, peripapillary retinoschisis has been found in glaucomatous eyes. In the meantime, retinoschisis may affect peripapillary RNFL measurement (peripapillary 3.4 mm circle), and caution is needed when comparing the peripapillary RNFL thickness obtained at different times to evaluate glaucomatous structural progression [4, 8].

To the best of our knowledge, and even after a review of the literature, there is no report on retinoschisis and DH coexisting in a glaucomatous eye. Therefore, there are several explanations for their coexistence. First, they may have been observed in the same eye by accident. Both are often found in glaucomatous eyes. Retinoschisis can occur in glaucoma patients, but may also occur without glaucoma. Even an association between retinoschisis and DH may be mere coincidence. Second, mechanical changes in the optic discs, such as LC defects, may share a common pathophysiology with these two phenomena [5, 9] However, no distinct anatomical change of the optic nerve has been found even with the currently available multimodal imaging. Hence, this could be a possible hypothesis.

One of the theories on the pathogenesis of DH is that capillary disruption occurs due to the predispositions to reactive gliosis, such as a maximum tractional force at the RNFL defect margin, the absence of an internal limiting membrane, and loose connective tissue support at the surface of the RNFL [2]. In these patients, retinoschisis occurs as part of the RNFL defect before DH occurs, and we can infer that retinoschisis may develop with these anatomical predispositions. A recent study showed that OCT signs of retinal glia (Müller) cell involvement could be associated with the pathogenesis of glaucoma-associated peripapillary retinoschisis [6]. Biomechanical forces on the optic nerve head are likely contributors to the development of peripapillary retinoschisis in glaucoma through the activation of mechanosensitive glia. Activated glial cells play an important role in the progression of glaucoma [7, 9, 10]. The progressive glaucomatous damage accompanying such glial dysfunction may induce peripapillary retinoschisis as an epiphenomenon of glaucoma progression [7]. From the perspective of this gliosis, DH and peripapillary retinoschisis may play similar roles in glaucoma.

Only one case does not provide sufficient information to provide a definitive explanation for the causal relationship between peripapillary retinoschisis and DH. As we did not continuously observe the changes in retinoschisis during the two follow-up visits, it is difficult to conclude that DH has a direct causal relationship with peripapillary retinoschisis. The case of DH following peripapillary retinoschisis in the same glaucomatous eye may demonstrate the possible pathophysiology and the relationship between these two phenomena in glaucoma.

## Data Availability

The datasets used and/or analysed during the current study are available from the corresponding author on reasonable request.

## Abbreviations

RNFL: Retinal nerve fiber layer
DH: Disc hemorrhage
LC: Lamina cribrosa
IOP: Intraocular pressure
OCT: Optical coherence tomography
